# Therapeutic Promises of Plant Metabolites against Monkeypox Virus: An In Silico Study

**DOI:** 10.1155/2023/9919776

**Published:** 2023-09-02

**Authors:** Anik Banik, Sheikh Rashel Ahmed, Sonia Binte Shahid, Tufayel Ahmed, Hafaza Khandaker Tamanna, Hlamrasong Marma

**Affiliations:** ^1^Department of Plant and Environmental Biotechnology, Sylhet Agricultural University, Sylhet 3100, Bangladesh; ^2^Faculty of Biotechnology and Genetic Engineering, Sylhet Agricultural University, Sylhet 3100, Bangladesh; ^3^Department of Horticulture, Sylhet Agricultural University, Sylhet 3100, Bangladesh

## Abstract

The monkeypox virus was still spreading in May 2022, with the first case identified in a person with travel ties to Nigeria. Using molecular docking-based techniques, we evaluated the efficiency of different bioactive chemicals obtained from plants against the monkeypox virus. A total of 56 plant compounds were evaluated for antimonekypox capabilities, with the top four candidates having a higher binding affinity than the control. We targeted the monkeypox profilin-like protein, which plays a key role in viral replication and assembly. Among the metabolites, curcumin showed the strongest binding affinity with a value of −37.43 kcal/mol, followed by gedunin (−34.89 kcal/mol), piperine (−34.58 kcal/mol), and coumadin (−34.14 kcal/mol). Based on ADME and toxicity assessments, the top four substances had no negative impacts. Furthermore, four compounds demonstrated resistance to deformability, which was corroborated by normal mode analysis. According to the bioactivity prediction study, the top compound target class was an enzyme, membrane receptor, and oxidoreductase. Furthermore, the study discovered that wortmannin, a gedunin analogue, can behave as an orthopoxvirus. The study found that these bioactive natural drug candidates could potentially work as monkeypox virus inhibitors. We recommended further experimental validation to confirm the promising findings of the study.

## 1. Introduction

The zoonotic monkeypox virus (MPXV), a type of orthopoxvirus, is native to western and central Africa. In the year of 1958, it was first identified and isolated from monkeys by the Statens Serum Institut of Copenhagen, Denmark [1]. The virus has been found in zoos and colonies of lab primates since then. MPXV falls into two distinct clades based on genetic, regional, and phenotypic variance, namely, west African and Congo Basin, with the latter's viruses being more virulent [2]. The MPX virus has a genome size of around 197 kbp length, similar to that of the smallpox variola (VAR) virus. However, it is not a progenitor or descendant of the VAR virus [3, 4].

Monkeypox is a zoonotic disease that causes clinical symptoms similar to smallpox in humans. The common symptoms include lymphadenopathy, fever, and rash, with swollen lymph nodes being the primary symptom that distinguishes it from smallpox and is seen in most patients before the appearance of a rash [5–8]. The recent MPX outbreak in 2022 has become a major concern to global health, as it is already affecting 19 countries across several continents [9]. Monkeypox's high potential for international spread and transmission make it an increasing concern. While the best methods for preventing and treating this potentially harmful disease are not yet known [10], two different forms of smallpox vaccines have been found to be effective against monkeypox. ACAM2000 is the most widely used vaccine and is approved in the US for smallpox protection. However, it should not be administered to at-risk groups, such as expectant or nursing mothers and individuals with compromised immune systems, due to potential adverse effects [11]. As the development and reemergence of highly contagious viruses continue to threaten global health, research into the antiviral activity of medicinal plants has accelerated considerably, aided by the growing availability of technological tools [12]. Medicinal plants contain a variety of biochemical and bioactive components that can be extracted and used to treat or prevent viral infections. Although the use of medicinal plants and natural products has been long known, scientific evidence and research into their preventive, therapeutic, and other health-related uses have only recently gained momentum. Through a range of scientific investigations, from identifying active ingredients to understanding the therapeutic mechanisms of antiviral herbs through clinical trials and their effective use in neutralizing viral infections, many herbs and plant metabolites have been screened, identified, and examined for their antiviral properties [13]. Computational methods, which use basic mathematical knowledge, have paved the way for a comprehensive understanding of newly emerging and reemerging infectious diseases along with pathogenesis, diagnosis, and treatment options where bioinformatics is indispensable to the discovery of novel drug and vaccine candidates against various viruses in a limited period of time [14].

## 2. Materials and Methods

### 2.1. Retrieval of Monkeypox Virus Key Protein and Plant Metabolites

To study potential antiviral metabolites against the monkeypox virus, we focused on the monkeypox profilin-like protein, which plays a key role in viral replication and assembly [15–17]. We retrieved the 3D structure of this protein (4qwo) from the RCSB Protein Data Bank. A total of 56 metabolites in the SDF format from various classes were obtained from the PubChem database using this method. These metabolites have been previously investigated for their antiviral properties and experimentally validated [18–72] (Supplementary File 1). PubChem is a database of chemical compounds and their responses to biological experiments [73]. We employed Open Babel v2.3 to change the metabolites' structure from the SDF format to the PDB format [74].

### 2.2. Molecular Docking of Antiviral Metabolites against Monkeypox Virus Profilin-Like Protein

We used molecular docking to evaluate the binding affinity of the 56 plant metabolites to the monkeypox profilin-like protein. We employed the PatchDock server to perform the docking process with the macromolecule small-ligand type and a clustering RMSD of 4.0 [75, 76]. The docking was performed with the help of the shape-based complementary principle of the docking algorithm which scans and allows to binds the small molecule into the binding pocket of the given macromolecule. The crystal PDB structure of protein molecules was prepared for docking by removing all water molecules and hetatms. To refine the docked complexes, the FireDock refinement tool was employed [77]. We used Discovery Studio for analysing the docking results [78]. Tecovirimat is a known inhibitor of monkeypox and used as a medicine for monkeypox infection, so we used it as a positive control [79]. The redocking of best candidates was performed though the Hdock server, which also refers that screened top drug candidates' binding affinity was stronger [80]. We also analysed the molecular interactions of tecovirimat with the monkeypox virus protein.

### 2.3. Drug Profile and Toxicity Analysis of Top Metabolites

We used the SwissADME server to assess their pharmacological features (absorption, distribution, metabolism, and excretion) [81]. The compounds were subjected to BOILED-Egg model analysis to determine their blood-brain penetration ability [82]. In addition to these, the pkCSM server was used to predict a number of toxic parameters such as LOAEL and LD_50_ [83].

### 2.4. Normal Mode Analysis

We employed normal mode analysis (NMA) to assess the conformational stability of the docked complex using the iMODS server [84]. The iMODS server elucidates data on the deformability, B-factors, and eigenvalues of the protein-ligand interactions to project the immanent motions' direction and size.

### 2.5. Prediction of Drug Targets and Available Drug Molecules from DrugBank

The study employed a tool—SwissTargetPrediction—to determine the expected target molecules for therapeutic candidates, hence confirming their bioactivity [85]. The server produced a collection of bioactive compounds at roughly 376,342 associated with around 3068 proteins. In addition, SwissSimilarity online tools were employed to identify current medication molecules with the potential for repurposing against monkey pox, using chemical or molecular similarity as the basis for screening [86].

## 3. Results

### 3.1. Screening of Plant Metabolites against Monkeypox Virus Profilin-Like Protein

**Figure 1:**
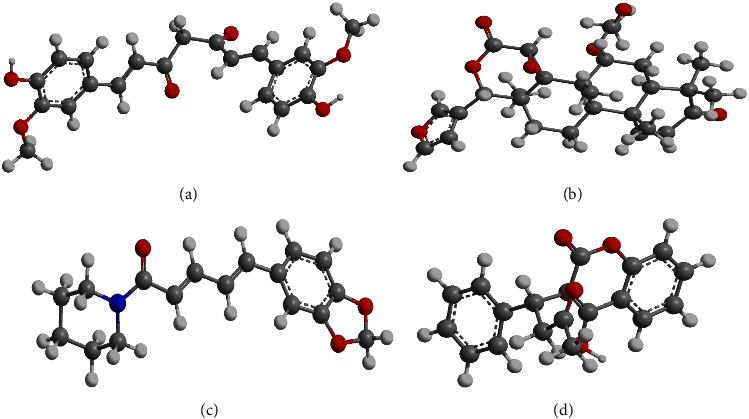
Chemical structures of curcumin (a), gedunin (b), piperine (c), and coumadin (d).

**Figure 2:**
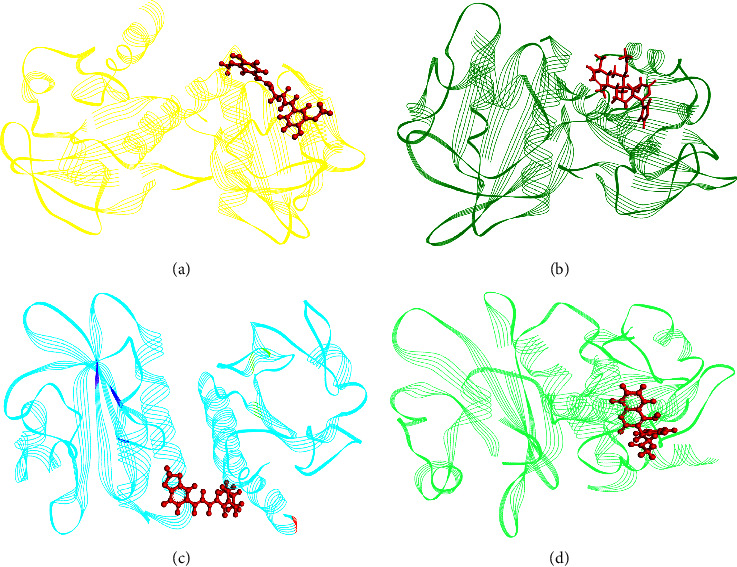
Molecular interaction of monkeypox virus profilin-like protein with curcumin (a), gedunin (b), piperine (c), and coumadin (d).

All of the downloaded structures of plant metabolites (ligands) and monkeypox protein (macromolecules) were optimized and used for docking purpose to determine the affinities between each ligands and macromolecule (Supplementary file 2). In each case, curcumin, gedunin, piperine, and coumadin (Figure 1) showed the best interactions with macromolecule (Figure 2 and Table 1). Moreover, curcumin displayed the strongest binding affinity with monkeypox protein (−37.43 kcal/mol), followed by gedunin (−34.89 kcal/mol), piperine (−34.58 kcal/mol), and coumadin (−34.14 kcal/mol), respectively. For the curcumin-4qwo complex, there were hydrogen bond, pi-pi stacked, carbon hydrogen, pi-pi T-shaped, pi-alkyl, and pi-donor hydrogen bond present. In the gedunin-4qwo complex, hydrogen bond, pi-donor hydrogen, and carbon hydrogen bond were present. Moreover, for the piperine-4wqo complex, hydrogen bond, alkyl, pi-pi T-shaped, and pi-alkyl bonds have been seen. Lastly, in the coumadin-4qwo complex, pi cation, pi-alkyl, and pi-pi stacked bonds were formed.

### 3.2. Analysis of the Drug Surface Hotspot and Ligand-Binding Pocket Prediction

**Figure 3:**
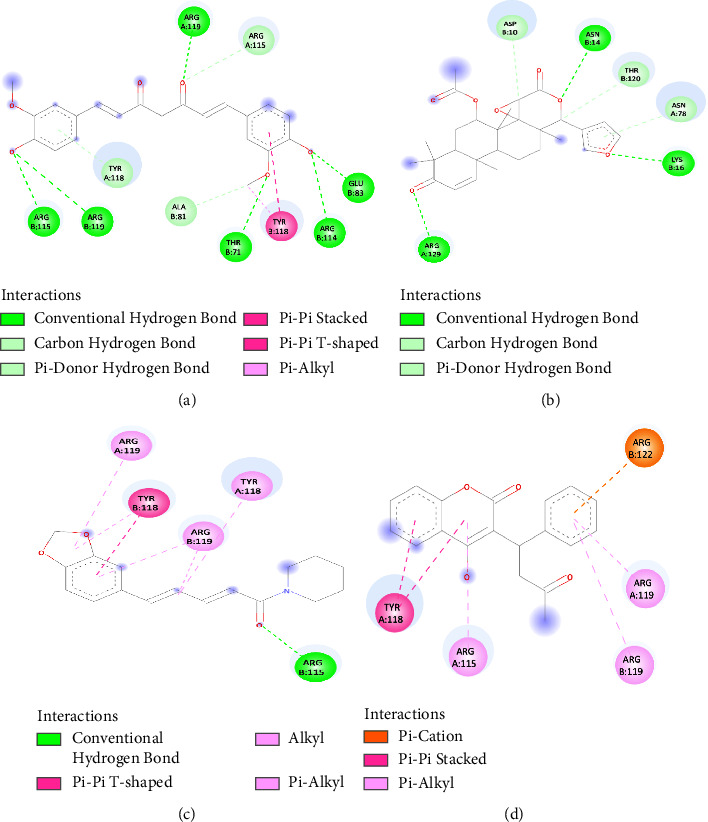
Drug surface hotspot of curcumin (a), gedunin (b), piperine (c), and coumadin (d).

The study investigated the structural structure of the docked complex to identify the drug surface hotspot of the targeted monkeypox proteins. We also examined the screened ligands' ligand-binding pattern and the interactions between the residues at each position (Figure 3, Table 1). Results showed that the binding interactions of the monkeypox protein were largely dependent on the amino acids at positions 78–129 for proteins chain A and 71–129 for proteins chain B. In most cases, the docked complexes were formed for both chains Arg 119, Arg115, and Tyr118.

### 3.3. ADME Analysis of Top Drug Candidates

**Figure 4:**
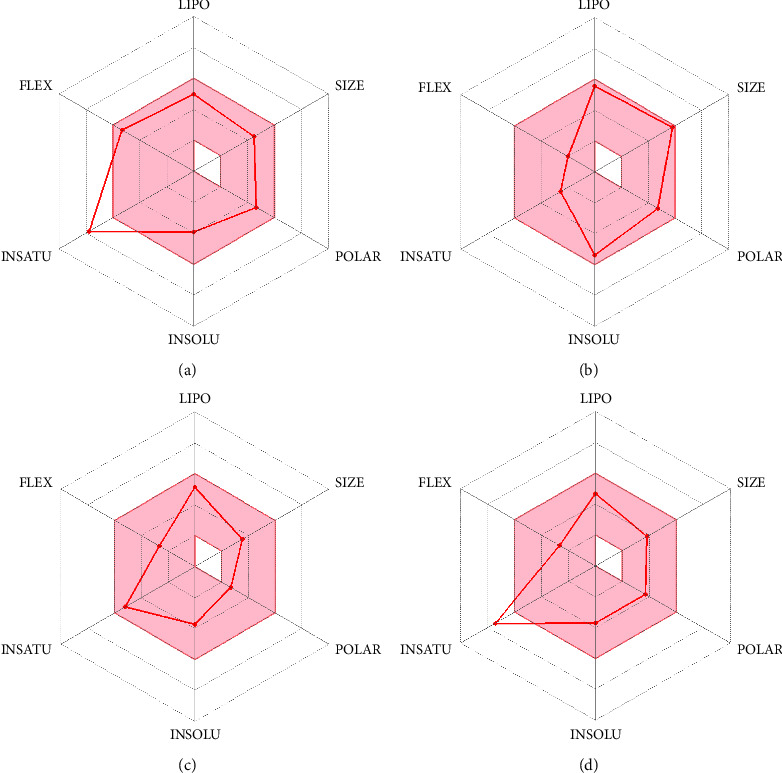
Screening of four metabolites with ADME analysis: curcumin (a), gedunin (b), piperine (c), and coumadin (d).

**Figure 5:**
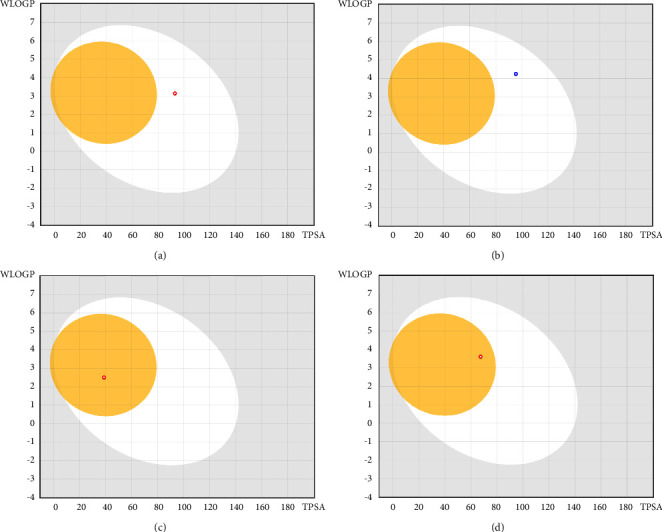
Analysis of proposed drug candidates with the BOILED-Egg model: curcumin (a), gedunin (b), piperine (c), and coumadin (d).

The drug profiles of our prioritized candidates were compared by calculating different ADME features (Table 2). The top four metabolites had greater GI absorption and positive interactions with several CYP isoforms. The optimal range for each property is as follows: lipophilicity: XLOGP3 between −0.7 and +5.0, size: MW between 150 and 500 g/mol, polarity: TPSA between 20 and 130 Å^2^, solubility: log *S* not higher than 6, and saturation and flexibility: no more than 9 rotatable bonds. All the compounds had LogP in between the range of −0.7 to +5.0 which refers to their lipophilicity and confirms their good absorption. The TPSA of each candidate was also in between the standard range which confirms their high intestinal absorption (Figure 4). All the compounds were found soluble or moderately soluble in each solubility parameters. Coumadin showed good solubility in two parameters except the log S (SILICOS-IT) parameter. All compounds have less than 10 rotatable bonds. All compounds were predicted orally bioavailable. But curcumin and coumadin were found a bit saturated in the bioavailability radar which might slower their absorption a bit. The BOILED-Egg model delivers a rapid, intuitive, easily reproducible yet statistically unprecedented robust method to predict the passive gastrointestinal absorption and brain access of small molecules useful for drug discovery and development. From the BBB study, gedunin and piperine showed they can pass the blood-brain barrier, so they have the potential to use for treating monkeypox causing complications in the brain (Figure 5).

### 3.4. Toxicity Pattern Analysis of Top Drug Candidates

Top drug candidates' skin sensitization, skin toxicity in minnows, skin toxicity in rats, and other toxicity criteria were anticipated (Table 3). Skin sensitization and AMES toxicity test results for candidate number four show negative findings. Top drug candidates produce negative results in the hERG I and hERG II inhibitor tests. The top four drug candidates were anticipated based on hepatotoxicity results to be liver safe. For a given compound, the maximum tolerated dose of less than or equal to 0.477 log (mol/kg/day) is considered low and high if greater than 0.477 log (mol/kg/day). All our top candidates were found lower than the value and refer the lower maximum tolerated dose. Oral rat acute toxicity at LD_50_ was found to be 1.833 for curcumin, 2.998 for gedunin, 1.773 for coumadin, and 2.811 for piperine, which means gedunin and piperine could be used in higher concentration for treatment than curcumin and coumadin. LOAEL values refer that curcumin can be used in the lowest dose for increased treatment lengths. *T. pyriformis*, with value >−0.5 log *μ*g/L, is considered toxic, and all the top candidates were found to be toxic to *T. pyriformis*. Minnow toxicity with value log LC50 <−0.3 is considered toxic for fathead minnows, where only curcumin was found to be toxic for fathead.

### 3.5. Normal Mode Analysis

**Figure 6:**
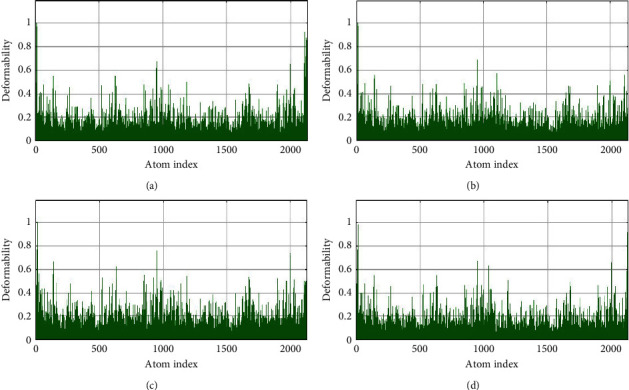
Deformability analysis of profilin-like protein with curcumin (a), gedunin (b), piperine (c), and coumadin (d).

**Figure 7:**
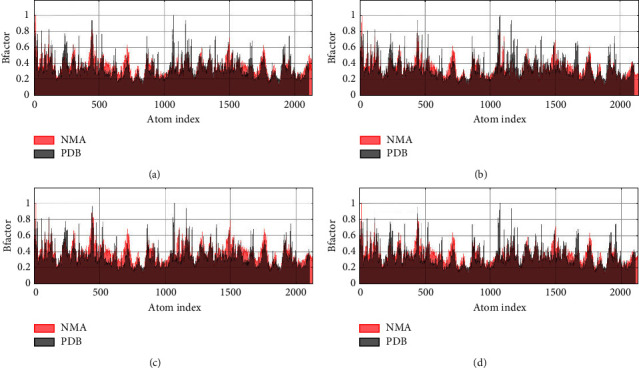
B-factor of profilin-like protein with curcumin (a), gedunin (b), piperine (c), and coumadin (d).

**Figure 8:**
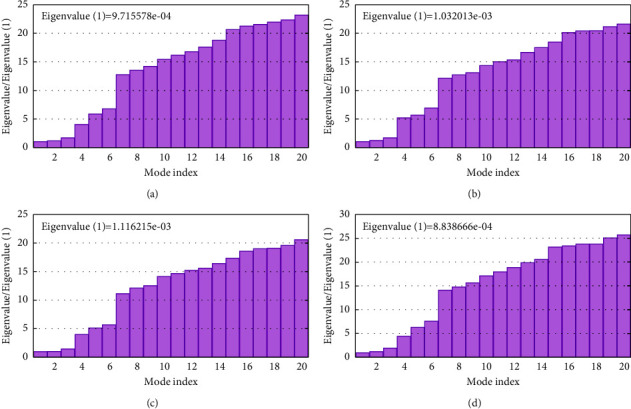
Eigenvalue of profilin-like protein with curcumin (a), gedunin (b), piperine (c), and coumadin (d).

**Figure 9:**
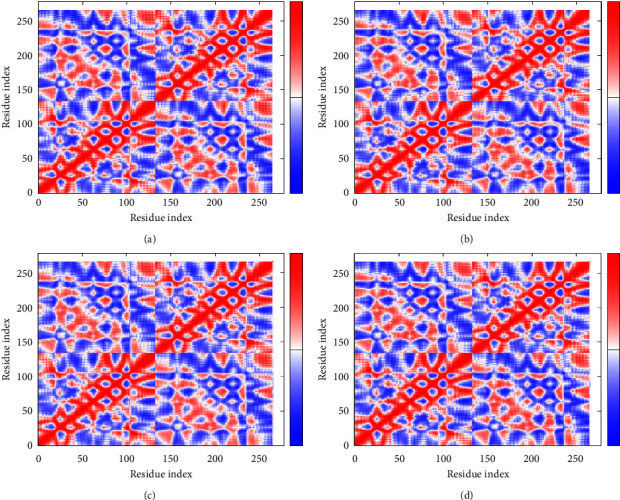
Covariance analysis of profilin-like protein with curcumin (a), gedunin (b), piperine (c), and coumadin (d).

The structure's hinges played a major role in how the structures deformed. All of the structure's hinges were not necessary and remained stable (Figures 6(a)–6(d)). The analysis of the B-factor showed that there were extremely few loop numbers and no significant changes (Figures 7(a)–7(d)). The stiffness of the motion is represented by the eigenvalue assigned to each normal mode. The structure's value is directly impacted by the quantity of energy required to deform it. The deformation is easier with the lower eigenvalue. The eigenvalues for the complexes were higher; the structure was compact, and it demonstrated its resistance to deformation. The eigenvalues were 9.715578 × 10^−4^ for the 4qwo-curcumin complex (Figure 8(a)), 1.032013 × 10^−3^ for the 4qwo-gedunin complex (Figure 8(b)), 1.116215 × 10^−3^ for the 4qwo-pirperine complex (Figure 8(c)), and 8.838666 × 10^−4^ for the 4qwo-coumadin complex (Figure 8(d)), respectively. The covariance matrix shows how closely two residue pairs are coupled, i.e., whether they move in correlated (red), uncorrelated (white), or anticorrelated (blue) ways (Figures 9(a)–9(d)).

### 3.6. Prediction of Drug Targets and Available Drug Molecules from DrugBank

**Figure 10:**
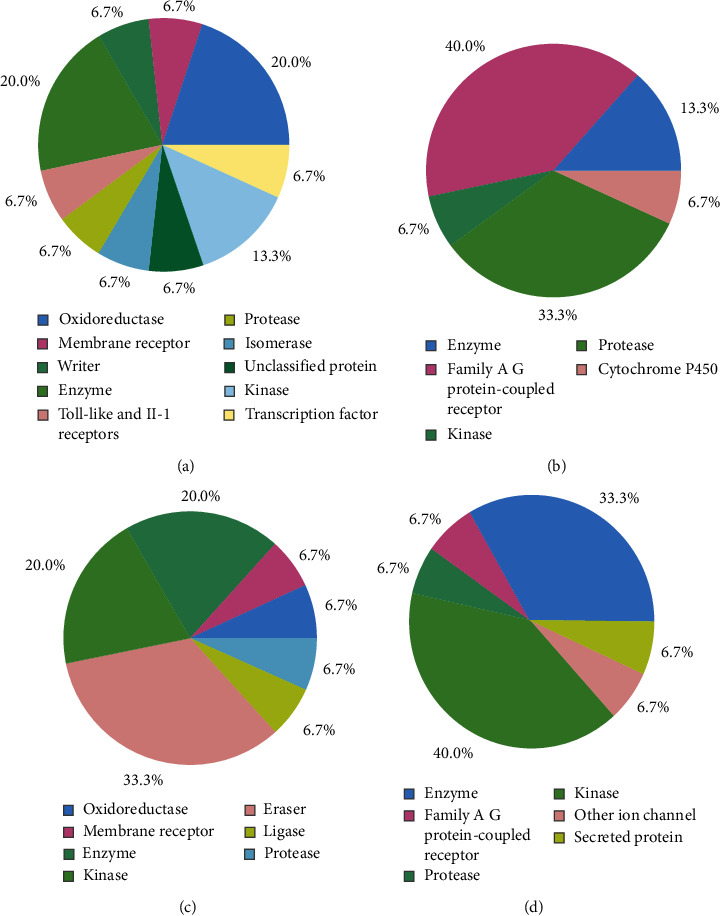
Predicted drug targets for curcumin (a), gedunin (b), piperine (c), and coumadin (d).

The majority of the target class belonged to membrane receptors, enzymes, and oxidoreductases (Figure 10, Table 4). To identify biologically active small molecules against the monkeypox virus from DrugBank, ligand-based virtual screening was carried out. With prediction scores of 0.694 and 0.658, respectively, two investigational medicines, ferulic acid (DB07767) and sinapic acid (DB12672), were discovered to be comparable to curcumin (Table 5).

## 4. Discussion

The disease brought on by the monkeypox virus (MPXV) can affect both people and animals. The majority of cases of human monkeypox, which clinically resembles common smallpox almost exactly, are discovered in the rainforests of central and western Africa [87]. In the summer of 2003, a well-known outbreak in the Midwest saw the first case of monkeypox disease in the western hemisphere and the United States. 37 of the 72 reported cases involving humans during an outbreak had their symptoms confirmed in a lab [88, 89]. Although the United States Federal Drug Administration recently licensed the drug tecovirimat, which is often effective against orthopoxviruses, it is also believed to be able to cure monkeypox [90] and was utilized as a positive control in this investigation. At the time, there was no approved treatment to treat variola virus infections although there were plant-derived natural chemicals that are significant because they provide a model molecule for the creation of new potential drugs [91]. Therefore, various plant-derived compounds were evaluated in the current investigation as potential inhibitors of the monkeypox virus protein by comparing their binding affinities to the essential protein of the pathogen. The speed of drug discovery has accelerated thanks to computational biology [92]. With global energies of −37.43 kcal/mol, 34.89 kcal/mol, −34.58 kcal/mol, and −34.14 kcal/mol, respectively, four plant metabolites—curcumin, gedunin, piperine, and coumadin—displayed better results in minimum binding energy than the control and other metabolites in this study. The results also illustrate that H-bonding and hydrophobic interactions are crucial for the stability of docked complexes [93, 94]. The structural conformation of the docked complexes was examined in light of the molecular docking results in order to identify the drug surface hotspot of our targeted monkeypox proteins, wherein the amino acids are at positions 78–129 for proteins chain A and 71–129 for proteins chain B. In most cases, Arg 119, Arg115, and Tyr118 were important binding sites. Therefore, the ADME study was performed on the top drug candidates to examine their pharmacological features. Any of our screened metabolites, however, did not display unintended effects that would have reduced their drug-like qualities. All the compounds had LogP in between the range of −0.7 to +5.0, which refers to their lipophilicity and confirms their good absorption. The TPSA of each candidate was also in between the standard range which confirms their high intestinal absorption. For the discovery of oral administrative drugs, solubility is one of the major descriptors [95]. All compounds have less than 10 rotatable bonds which are in favor of binding to their target to avoid entropic penalty [96]. The top four metabolites showed greater GI absorption. Analysis of the inhibitory effects with several CYP isoforms showed positive interactions between the CYP isoform and top candidates. MPXV not only can cause long-lasting brain injury but also can induce other neurological manifestations [97]. From the BBB study, gedunin and piperine showed they can pass the blood-brain barrier, so they have potential to use for treating monkeypox causing complications in the brain. Each candidate might be soluble in water. Skin sensitization and the AMES toxicity test on the four tested candidate yield negative findings. Top drug candidates showed negative outcome in hERG I inhibitors and hERG II inhibitors. The hepatotoxicity result predicted that the top four drug candidates were safe for the liver. Maximum-tolerated dose (human) values were low for top drug candidates. This indicated that toxicity of the top four drugs candidates was good and that they did not show any undesirable properties. In the NMA study, deformability of the protein-ligand complex revealed that the structure was resistant to deformation and had higher eigenvalues, supporting our claim that complexes exhibit resistance to deformation and maintain stability (Figure 8). The B-factor analysis finds no significant fluctuation. The drug target analysis of the target class belonged to oxidoreductases, membrane receptors, and enzymes. Due to its huge size, 350 × 270 nm, the Orthopoxvirus-Vaccinia virus was the zoonotic virus to be visible easily under a microscope. According to a recent report, its dsDNA genome (around 200 kbp) has the capacity to encode about 209 gene products [98]. The results of a more thorough investigation into the vaccinia virus assist to activate the MEK/ERK pathway, confirming that the signals generated by the virus-host contact stimulated downstream targets, ribosomal S6 kinase 2, and ternary complex factor Elk-1, which in turn caused the production of early growth response factor-1 (EGR-1). VGF is necessary for the maintenance of the active MEK/ERK/RSK2/Elk-1/EGR-1 pathway, and its disruption by pharmacological inhibition or genetic ablation drastically reduced the virus production [99]. Coumadin can show bioactivity against MAP kinase-ERK2 (Table 4), and the major enzyme groups were the bioactive targets for the other top metabolites (Figure 10). The Orthopoxvirus family includes the monkeypox virus. Therefore, it is possible for the top 4 metabolites to reduce the monkeypox virus production by interfering these enzymatic pathways. Sinapic acid and dicoumarol, two approved structural analogues of ferulic acid, were discovered by the drug similarity prediction as potential alternatives and, as a result, need additional in vivo research. Wortmannin from DrugBank was one of the biologically active compounds that were predicted by ligand-based virtual screening utilizing gedunin.

## 5. Conclusion

The analysis of ADME (absorption, distribution, metabolism, and excretion) showed that our proposed bioactive drug candidates have properties that make them suitable for drug use. Furthermore, the toxicity study revealed that curcumin, gedunin, piperine, and coumadin, the four compounds under investigation, did not cause any harmful effects. These findings indicate that these natural chemicals have the potential to act as inhibitors of the monkeypox virus. Although the results are promising, we strongly recommend further testing in living organisms to validate these experimental findings.

## Figures and Tables

**Table 1:** Docking result and the binding site of our top ranked plant-derived metabolites against monkeypox profilin-like protein (including tecovirimat, the control used in the study).

Macromolecules	Ligands	Global energy (kcal/mol)	HdocK docking score	Score	Area	ACE	Binding site
Profilin-like protein (4qwo)	Curcumin	−37.43	−175.62	4394	488.50	−9.18	Arg119a, Arg115a, Glu83b, Arg114b, Tyr118a, Thr71b, Ala81b, Arg115b, Arg119b
	Gedunin	−34.89	−176.00	5432	608.40	1.26	Asp10b, Asn14b, Asn78a, Thr120b, Lys16b, Arg129a
	Piperine	−34.58	−141.14	3812	463.60	−9.82	Arg119a, Tyr118a, Arg115b, Tyr118b, Arg119b
	Coumadin	−34.14	−161.58	3748	446.90	−9.21	Arg119a, Tyr118a, Arg122b, Arg115a, Arg119b
	Tecovirimat (control)	−20.41	−140.38	4162	469.00	1.79	Asp116a, Thr120a, Arg119a, Asn 78b, Arg129b, His100b

**Table 2:** Drug profile and ADME analysis of top-ranked metabolites.

Parameters	Curcumin	Gedunin	Piperine	Coumadin
Molecular weight (g/mol)	368.38	482.57	285.34	308.33
TPSA (Å^2^)	93.06	95.34	38.77	67.51
Log Po/w (iLOGP)	3.27	3.19	3.38	2.41
Log Po/w (WLOGP)	3.15	4.24	2.51	3.61
Log Po/w (SILICOS-IT)	4.04	4.44	3.41	4.36
Gastrointestinal absorption	High	High	High	High
BBB permeant	No	No	Yes	Yes
P-gp substrate	No	Yes	No	No
CYP1A2 inhibitor	No	No	Yes	No
CYP2C19 inhibitor	No	No	Yes	Yes
CYP2C9 inhibitor	Yes	No	Yes	Yes
CYP2D6 inhibitor	No	No	No	No
CYP3A4 inhibitor	Yes	No	No	No
Log S (ESOL)	−3.94	−5.40	−3.74	−3.70
Solubility in mg/ml	4.22*e − *02	1.93*e − *03	5.24*e − *02	6.10*e − *02
Class	Soluble	Moderately soluble	Soluble	Soluble
Log S (Ali)	−4.83	−5.93	−3.96	−3.77
Solubility in mg/ml	5.50*e − *03	5.64*e − *04	3.16*e − *02	5.23*e − *02
Class	Moderately soluble	Moderately soluble	Soluble	Soluble
Log S (SILICOS-IT)	−4.45	−5.75	−3.00	−6.33
Solubility in mg/ml	1.31*e − *02	8.50*e − *04	2.87*e − *01	1.45*e − *04
Class	Moderately soluble	Moderately soluble	Soluble	Poorly soluble
Hydrogen-bond acceptors	6	7	3	4
Hydrogen-bond donors	2	0	0	1
Total rotatable bonds	8	3	4	4

**Table 3:** Toxicity properties analysis of screened drug candidates.

Toxicity properties	Curcumin	Gedunin	Piperine	Coumadin
AMES toxicity	−	−	−	−
Maximum tolerated human dose	0.081	−0.736	−0.38	0.294
hERG I inhibitor	−	−	−	−
hERG II inhibitor	−	−	−	−
Oral rat Acute toxicity at LD_50_ (mg/kg_bw/day)	1.833	2.998	2.811	1.773
Oral rat chronic toxicity at LOAEL	2.228	0.195	1.51	1.081
Hepatotoxicity	−	−	+	−
Skin sensitisation	−	−	−	−
Toxicity in *T. pyriformis*	0.494	0.291	1.879	0.591
Minnow toxicity	−0.081	0.456	1.732	0.034

**Table 4:** List of drug targets for curcumin, coumadin, piperine, and gedunin.

Metabolites	Drug targets	Common name	Target class	Probability
Curcumin	MAO (monoamine oxidase A)	MAO-A	Oxidoreductase	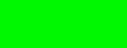
	Beta amyloid A4 precursor protein	APP	Membrane receptor	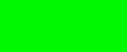
	Histone acetyltransferase p300	EP300	Writer	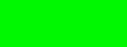
	Prostaglandin E synthase	PTGES	Enzyme	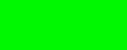

Coumadin	MAP kinase ERK2	MAPK1	Kinase	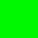
	Aldose reductase	AKR1B1	Enzyme	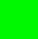

Piperine	Monoamine oxidase B	MAOB	Oxidoreductase	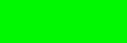
	Sigma opioid receptor	SIGMAR1	Membrane receptor	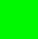

Gedunin	Macrophage migration inhibitory factor	MIF	Enzyme	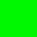
	Kappa opioid receptor	OPRK1	Family A G protein-coupled receptor	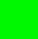
	Delta opioid receptor	OPRD1	Family A G protein-coupled receptor	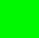

**Table 5:** Predicted structural analogue molecules from DrugBank.

Metabolites	Name	Status	Score	DrugBank Id
Curcumin	Ferulic acid	Experimental	0.694	DB07767
	Sinapic acid	Experimental	0.658	DB08587

Coumadin	Dicoumarol	Approved	0.755	DB00266
	Ethyl biscoumacetate	Withdrawn	0.646	DB08794

Gedunin	Wortmannin	Experimental	0.488	DB08059

## Data Availability

The data that support the findings of this study are included within the article and its supplementary materials.

## Abbreviations

MPV: Monkeypox virus
ADME: Absorption, distribution, metabolism, and excretion
NMA: Normal mode analysis.

## Supplementary Materials

Supplementary Materials Supplementary File 1: list of 56 antiviral plant metabolites obtained from literature studies. Supplementary File 2: docking results of plant-derived metabolites against monkeypox profilin-like protein.
