# Supplementary material for: Therapeutic Promises of Plant Metabolites against Monkeypox Virus: An In Silico Study
Source: Adv Virol. 2023 Sep 2;2023:9919776. doi: 10.1155/2023/9919776 (PMC10492655; doi:10.1155/2023/9919776)
Supplement: Supplementary Materials — Supplementary File 1: list of 56 antiviral plant metabolites obtained from literature studies. Supplementary File 2: docking results of plant-derived metabolites against monkeypox profilin-like protein. [file 9919776.f1.zip › Supplementary File 1 (1).docx]

Supplementary File 1: List of 56 antiviral plant metabolites obtained from literature studies

| ***Metabolites*** | ***Pubchem id*** | ***Chemical Class*** | ***Plant Source*** | ***Molecular Formula*** | ***Reference*** |
| --- | --- | --- | --- | --- | --- |
| Allicin | 65036 | Thiosulfinate | *Allium sativum* | C6H10OS2 | [18] |
| Curcumin | 969516 | Diarylheptanoid | *Curcuma longa* | C21H20O6 | [19] |
| Quercetine | 5280343 | Flavonoid | *Allium cepa* | C15H10O7 | [20] |
| Asparagine | 6267 | Organic compounds | *Citrus aurantifolia* | C4H8N2O3 | [21] |
| Phyllanthin | 358901 | Bioactive lignan | *Phyllanthus amarus* | C24H34O6 | [22] |
| Naphthoquinoneimine | 67449396 | Naphthoquinones | *Kigelia pinnata* | C10H7NO2 | [23] |
| Citreorosein | 361512 | Hydroxyanthraquinones | *Polygonum cuspidatum* | C16H12O6 | [24] |
| Emodin | 3220 | Trihydroxyanthraquinone | *Reynoutria japonica* | C15H10O5 | [25] |
| Pyrenocine A | 6312351 | Pyrans | *Penicillium paxilli* | C11H12O4 | [26] |
|  |  |  |  |  |  |
| Harzianopyridone | 54697782 | Pyrans | *Trichoderma harzianum* | C14H19NO5 | [27] |
| Fuscinarin | 10445499 | Gallic acid | *Aegiceras corniculatum* | C14H16O5 | [28] |
| Allyl propyl | 16591 | Allyl sulfur compounds | *Allium cepa* | C6H12S2 | [29] |
| disulfide |  |  |  |  |  |
| Apigenin | 5280443 | Flavonoids | *Passiflora foetida* | C15H10O5 | [30] |
| Artocarpesin | 399491 | 6-prenylated | *Artocarpus* | C20H18O6 | [31] |
|  |  | flavones | *heterophyllus* |  |  |
| Ascorbic acid | 54670067 | Dihydrofurans | *Citrus aurantifolia* | C6H8O6 | [32] |
| Citronellal | 7794 | Prenol lipids | *Cymbopogon* | C10H18O | [33] |
|  |  |  | *citrates* |  |  |
| Coumadin | 54678486 | Coumarins and derivatives | *Sida acuta Burm* | C19H16O4 | [34] |
| Gingerol | 442793 | Phenols | *Zingiber officinale* | C17H26O4 | [35] |
| Limonin | 179651 | Prenol lipids | *Cymbopogon* | C26H30O8 | [36] |
|  |  |  | *citrates* |  |  |
| Norartocarpetin | 5481970 | Flavonoids | *Artocarpus* | C15H10O6 | [37] |
|  |  |  | *heterophyllus* |  |  |
| Quinine | 3034034 | Cinchona alkaloids | *Azadirachta indica* | C20H24N2O2 | [38] |
| Riboflavin | 493570 | Pteridines and | *Moringa oleifera* | C17H20N4O6 | [39] |
|  |  | derivatives |  |  |  |
| Stigmasterol | 5280794 | Steroids and | *Amaranthus* | C29H48O | [40] |
|  |  | steroid derivatives | *spinosus* |  |  |
| Triterpenoids | 71597391 | Prenol lipids | *Sida acuta Burm* | C29H44O5 | [41] |
| Vanillin | 1183 | Phenols | *Vanilla planifolia* | C8H8O3 | [42] |
| Vitexin | 5280441 | Apigenin flavone glucoside | *Crataegus species* | C21H20O10 | [43] |
| Andrographolide | 5318517 | Diterpenoid labdane | *Andrographis paniculata* | C20H30O5 | [44] |
| Capsaicin | 1548943 | Alkaloid | *Capsicum annuum* | C18H27NO3 | [45] |
| Cinnamic acid | 444539 | Aromatic carboxylic acids | *Cinnamomum species* | C9H8O2 | [46] |
| Eugenol | 3314 | Phenylpropanoid | *Ocimum tenuiflorum, Eugenia caryophyllata* | C10H12O2 | [47] |
| Galangin | 5281616 | Flavonol | *Helichrysum aureonitens* | C15H10O5 | [48] |
| Kaempferol | 5280863 | Flavonoid aglycone | *Capparis spinose* | C15H10O6 | [49] |
| Luteolin | 5280445 | Flavonoid | *Carrots, celery peppers, olive peppermint* | C15H10O6 | [50] |
| Piperine | 638024 | Alkaloid | *Piper spp.* | C17H19NO3 | [51] |
| Thymoquinone | 10281 | Monoterpene | *Nigella sativa* | C10H12O2 | [52] |
| Guaiol | 227829 | Sesquiterpenoid | *Piper nigrum* | C15H26O | [53] |
| Mangiferin | 5281647 | Organic compounds xanthones | *Mangifera indica* | C19H18O11 | [54] |
| Gallic acid | 370 | Phenolic acid | *Arctostaphylos uva-ursi* | C7H6O5 | [55] |
| Fenchone | 14525 | A monoterpenoid and a ketone | *Foeniculum vulgare* | C10H16O | [56] |
| Cuminaldehyde | 326 | Aromatic monoterpenoids | *Cuminum cyminum* | C10H12O | [57] |
| Gamma-Terpinene | 7461 | Menthane monoterpenoids | *Melaleuca alternifolia* | C10H16 | [58] |
| p-Cymene | 7463 | Alkylbenzene | *Chrysophillum albidum* | C10H14 | [59] |
| Hesperetin | 72281 | Flavonoid | *Citrus limon* | C16H14O6 | [60] |
| Fisetin | 5281614 | Flavonoid | *Fragaria ananassa* | C15H10O6 | [61] |
| Myricetin | 5281672 | Flavonoid | *Rosa canina* | C15H10O8 | [62] |
| Hesperidin | 10621 | Flavanone glycoside | *Citrus medica* | C28H34O15 | [63] |
| Naringenin | 932 | Flavanones | *Citrus paradisi* | C15H12O5 | [64] |
| Adenine | 190 | 6-aminopurines | *Phaseolus vulgaris* | C5H5O5 | [65] |
| Zingerone | 31211 | Methoxyphenols | *Zingiber officinale* | C11H14O3 | [66] |
| Anisotine | 442884 | Alkaloids | *Justicia adhatoda* | C20H19N3O3 | [67] |
| Oleanolic acid | 10494 | Triterpenoids | *Olea europaea* | C30H48O3 | [68] |
| Coumestan | 638309 | Heterocyclic organic compound | *Eclipta alba* | C15H8O3 | [69] |
| Humulene epoxide | 5352470 | Epoxides | *Cyperus rotundus* | C15H24O | [70] |
| Rosmarinic acid | 5281792 | Phenolic compound and ester of caffeic acid | *Rosmarinus officinalis* | C18H16O8 | [71] |
| Ajoene | 5386591 | Organosulfur compound | *Allium sativum* | C9H14OS3 | [72] |
| Gedunin | 12004512 | Limonoids | *Azadirachta indica* | C28H34O7 | [73] |
