# Supplementary material for: Therapeutic Promises of Plant Metabolites against Monkeypox Virus: An In Silico Study
Source: Adv Virol. 2023 Sep 2;2023:9919776. doi: 10.1155/2023/9919776 (PMC10492655; doi:10.1155/2023/9919776)
Supplement: Supplementary Materials — Supplementary File 1: list of 56 antiviral plant metabolites obtained from literature studies. Supplementary File 2: docking results of plant-derived metabolites against monkeypox profilin-like protein. [file 9919776.f1.zip › Supplementary File 2 (1).docx]

Supplementary File 2: Docking results of plant derived metabolites against monkeypox profilin like Protein

| ***Macromolecules*** | ***Metabolites Name*** | ***Global Energy*** | ***ACE*** | ***Score*** | ***Area*** |
| --- | --- | --- | --- | --- | --- |
| Profilin like Protein (4qwo) | Allicin | -27.82 | -9.38 | 2616 | 277.70 |
|  | Curcumin | -37.43 | -9.18 | 4394 | 488.50 |
|  | Quercetine | -26.29 | -1.41 | 3518 | 377.20 |
|  | Asparagine | -16.47 | 5.54 | 2036 | 213.50 |
|  | Phyllanthin | -29.85 | -3.81 | 4980 | 556.40 |
|  | Naphthoquinoneimine | -19.61 | -0.82 | 2702 | 289.20 |
|  | Citreorosein | -26.48 | 0.40 | 3782 | 422.20 |
|  | Emodin | -26.24 | -0.58 | 3480 | 393.90 |
|  | Pyrenocine A | -23.48 | -0.43 | 3378 | 385.20 |
|  | Harzianopyridone | -23.05 | -1.41 | 3902 | 466.30 |
|  | Fuscinarin | -22.61 | -0.66 | 3420 | 393.40 |
|  | Allyl Propyl Disulfide | -25.76 | -9.53 | 2426 | 276.20 |
|  | Apigenin | -27.41 | -2.04 | 3490 | 370.20 |
|  | Artocarpesin | -27.98 | -0.34 | 4844 | 529.90 |
|  | Ascorbic Acid | -19.10 | -4.44 | 2298 | 249.90 |
|  | Citronellal | -21.15 | -2.20 | 3514 | 365.30 |
|  | Coumadin | -34.14 | -9.21 | 3748 | 446.90 |
|  | Gingerol | -21.93 | -0.99 | 3834 | 448.00 |
|  | Limonin | -27.63 | -3.89 | 4580 | 521.90 |
|  | Norartocarpetin | -23.29 | -1.38 | 3334 | 374.00 |
|  | Quinine | -27.12 | -1.51 | 4152 | 508.80 |
|  | Riboflavin | -31.28 | -3.00 | 4564 | 544.00 |
|  | Stigmasterol | -21.02 | 0.10 | 4900 | 657.60 |
|  | Triterpenoids | -32.18 | -5.80 | 4682 | 539.00 |
|  | Vanillin | -16.61 | -0.71 | 2538 | 268.50 |
|  | Vitexin | -28.69 | 0.22 | 4104 | 453.70 |
|  | Andrographolide | -28.76 | -1.62 | 4006 | 452.90 |
|  | Capsaicin | -26.99 | -1.55 | 4174 | 458.30 |
|  | Cinnamic Acid | -19.39 | -1.84 | 2752 | 282.20 |
|  | Eugenol | -26.38 | -2.26 | 3096 | 331.50 |
|  | Galangin | -27.14 | -3.07 | 3352 | 390.40 |
|  | Kaempferol | -26.41 | -1.82 | 3674 | 385.10 |
|  | Luteolin | -27.55 | -1.75 | 3386 | 363.80 |
|  | Piperine | -34.58 | -9.82 | 3812 | 463.60 |
|  | Thymoquinone | -23.17 | -6.01 | 2850 | 297.40 |
|  | Guaiol | -26.06 | -3.31 | 3584 | 389.00 |
|  | Mangiferin | -26.17 | 2.74 | 4228 | 476.50 |
|  | Gallic Acid | -20.30 | -4.91 | 2342 | 262.90 |
|  | Fenchone | -22.82 | -6.63 | 2252 | 236.30 |
|  | Cuminaldehyde | -21.64 | -6.03 | 2524 | 275.00 |
|  | Gamma-Terpinene | -19.40 | -4.93 | 2528 | 302.40 |
|  | P-Cymene | -18.31 | -1.68 | 2934 | 321.00 |
|  | Hesperetin | -24.56 | -0.75 | 4110 | 442.80 |
|  | Fisetin | -30.00 | -9.47 | 3474 | 383.40 |
|  | Myricetin | -24.22 | -1.91 | 3474 | 381.90 |
|  | Hesperidin | -28.03 | 1.13 | 5580 | 680.60 |
|  | Naringenin | -24.98 | -0.41 | 3628 | 415.50 |
|  | Adenine | -20.51 | -6.67 | 2080 | 230.50 |
|  | Zingerone | -22.91 | -1.66 | 3386 | 352.10 |
|  | Anisotine | -29.35 | -2.46 | 4518 | 528.20 |
|  | Oleanolic Acid | -27.52 | -1.89 | 5002 | 584.70 |
|  | Coumestan | -31.35 | -8.60 | 3260 | 360.70 |
|  | Humulene Epoxide | -25.40 | -6.64 | 3314 | 353.20 |
|  | Rosmarinic Acid | -32.99 | -5.39 | 4198 | 485.30 |
|  | Ajoene | -27.95 | -6.70 | 3584 | 392.40 |
|  | Gedunin | -34.89 | 1.26 | 5432 | 608.40 |
